# Long-term outcome of Non-Transecting Anastomotic Bulbar Urethroplasty for Urethral Strictures: An 8-year experience from Liaquat National Hospital Karachi

**DOI:** 10.12669/pjms.37.5.3879

**Published:** 2021

**Authors:** Aziz Abdullah, Syed Farhan Ahmed, Imran Idrees Memon

**Affiliations:** 1 Prof. Aziz Abdullah, FRCS, FEBU. Department of Urology, Liaquat National Hospital & Medical College Karachi, Pakistan; 2 Dr. Syed Farhan Ahmed, FCPS. Department of Urology, Liaquat National Hospital & Medical College Karachi, Pakistan; 3 Dr. Imran Idrees Memon, FCPS. Ex-Registrar, Liaquat National Hospital, Karachi, Pakistan. Department of Urology, Liaquat University of Medical & Health Sciences Jamshoro, Sindh, Pakistan

**Keywords:** Non-transecting, bulbar urethra, anastomotic urethroplasty, Stricture, Urethroplasty, Complications, Outcome, end-to-end urethroplasty

## Abstract

**Objectives:**

To determine the long-term outcome and complications of non-transecting anastomotic bulbar urethroplasty for the treatment of small bulbar urethral strictures presenting at Liaquat National Hospital, Karachi.

**Methods:**

This interventional study was conducted from January 2012 to December 2019 with the study duration of eight year at Liaquat National Hospital, Karachi. All patients presenting in the outpatient department with urethral strictures were included in the study. Patients were evaluated postoperatively for complications and outcomes were determined. The data was analyzed using SPSS v.25.

**Results:**

A total of 358 patients were treated with non-transecting anastomotic bulbar urethroplasty during this 8-years period. The most common site of stricture formation was bulbar urethra 186 (50%), followed by bulbo-membranous urethra; 103 (31%), and bulbo-penile urethra; 69 (19%). The mean stricture was 1.2 cm (0.5-2.5 cm) in length. The main postoperative complications were scrotal swelling in 7 (1.9%), wound infection in 6 (1.6%), wound dehiscence in 3 (0.8%), and transient sexual dysfunction in 31 (8.6%) patients with an overall initial success rate of 97.8%. No permanent deficit in sexual function was reported.

**Conclusions:**

Non-transecting anastomotic bulbar urethroplasty has a good outcome with insignificant postoperative complications in patients with small bulbar urethral stricture disease.

## INTRODUCTION

The bulbar urethra is the commonest site for urethral strictures in the developing world. The commonest causes of these strictures are idiopathic followed by trauma. Most common site of these strictures is at the junction of the proximal and middle thirds. These short, sharp strictures in this location may be congenital in origin due to failure of complete canalization of the urogenital membrane.^1^

The surgical treatment of urethral strictures depends on the cause, length, and the location of the stricture.^2^ Previously, the best initial treatment was considered to be the endoscopic instrumentation by dilatation or urethrotomy. However, due to high recurrence and massive scar formation, followed by post-traumatic strictures formation it has now been replaced with non-transecting anastomotic urethroplasty and other reconstructive surgeries.^3^

Currently urethroplasty is the only main curative option for short bulbar strictures. However, only specialized surgeons can do this surgery.^4^ Non-transecting anastomotic urethroplasty has been the preferred urethroplasty technique for short bulbar strictures and is associated with an excellent functional outcome and a low complication rate.^5^

The present study is an extension to the previously published paper on the 2-year long experience of non-transecting anastomotic urethroplasty. In this study, we present a 8-year long experience of bulbar stricture disease surgically treated with non-transecting anastomotic bulbar urethroplasty with a primary focus on the outcome and postoperative complications.

In the present study, we have reported the long-term outcome and complications of non-transecting anastomotic urethroplasty for the treatment of urethral strictures presenting at Liaquat National Hospital, Karachi.

## METHODS

This was a prospective interventional study conducted at Liaquat National Hospital, Karachi from January 2012 to December 2019. After obtaining ethical approval from the Institutional Review Board Committee (Reference # LNH/687/2012), a total of 358 patients were treated with non-transecting anastomotic bulbar urethroplasty during this 8-year period. All patients presenting in the outpatient department with lower urinary tract symptoms (LUTS) including voiding or obstructive symptoms such as hesitancy, poor or intermittent stream, dribbling, and storage symptoms such as frequency, urgency, incontinence, and nocturia with diagnostic evidence of a stricture were included in the study. Patients with strictures other than at the bulbo-penile junction, bulbar urethra, and bulbo-membranous urethra were excluded from the study. A stricture of less than one cm to 2.5 cm was considered eligible for surgical treatment with non-transecting anastomotic bulbar urethroplasty.

After obtaining informed written consent, patient history & examination was done. Preoperative evaluation was done prior to surgery for all participants. Preoperative evaluation included renal function tests, urine analysis, U/S KUB & post void residual, uroflowmetry, ascending & descending urethrogram, and other routine laboratory investigations. Antibiotic prophylaxis with a third generation cephalosporins were prescribed to all patients. Deep Venous Thrombosis (DVT) prophylaxis was provided, if indicated. Patients were evaluated postoperatively and on each follow-up visits on the 2nd, 8th and 24th week postoperatively. The data was analyzed using Statistical Package for the Social Sciences (SPSS v.25).

## RESULTS

**Fig.1:**
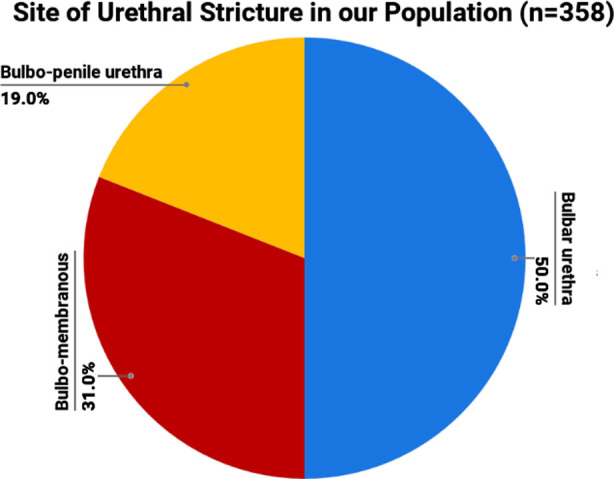
Site of Bulbar Urethral Strictures in our Population (n=358).

A total of 358 patients were treated for bulbar strictures with non-transecting anastomotic urethroplasty. The mean age ± SD of patients were 38 ± 13.5 years with a range of 20-70 years. The most common site of stricture formation was bulbar urethra 186 (50%), followed by bulbo-membranous urethra; 103 (31%), and bulbo-penile urethra; 69 (19%) (Fig.1). The most common cause of stricture formation was idiopathic 154 (43%), followed by iatrogenic injuries 82 (23%), trauma 78 (22%), and inflammatory causes 44 (12%). Table-I.

**Table-I:** Demographical and Clinical Profile of Patients with Urethral Stricture Disease (n=358).

Variable	Frequency n (%)
Mean Age ± SD (in years)	38 ± 13.5 years
***Stricture Site***	
Bulbar urethra	179 (50%)
Bulbo-membranous urethra	111 (31%)
Bulbo-penile urethra	68 (19%)
***Etiology***	
Idiopathic	154 (43%)
Trauma	78 (22%)
Iatrogenic injury	82 (23%)
UTI/Inflammatory	44 (12%)
Mean Stricture Length	1.2 cm (0.5cm - 2.5cm)
Mean Follow up Duration	12 months (6 months - 3years)
Overall Success Rate	97.8%
***Operative findings and success rate of patients treated with Non-transecting Anastomotic Bulbar Urethroplasty***	
Mean Operative Duration ± SD (in minutes)	41 + 12.65 minutes
Intraoperative blood loss	<100ml
Intraoperative complications	Nil
***Postoperative complications***	
Scrotal swelling	7 (1.9%)
Wound Infection	6 (1.6%)
Wound Dehiscence	3 (0.8%)
Transient Erectile Dysfunction	31 (8.6%)
Permanent Erectile Dysfunction	0
Procedure Failure	0
Initial Success Rate	97.8%

The mean operative duration ± SD were reported to be 41 ± 12.65 minutes. The intraoperative blood loss was approximately 100 ml among the patients who were selected for the surgery Table-I.

**Fig.2:**
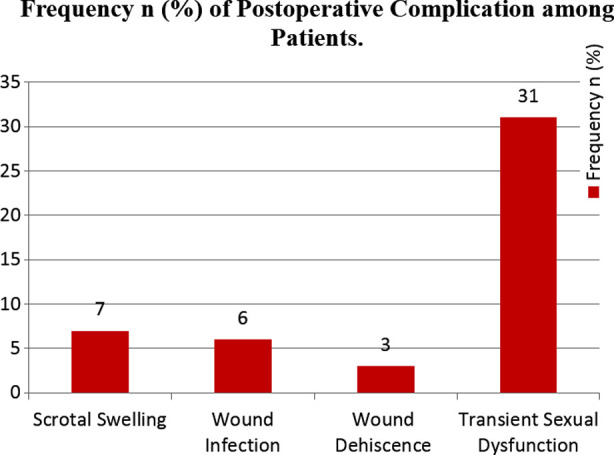
Postoperative complications among patients who underwent Non-transecting Anastomotic Bulbar Urethroplasty.

The main post-surgical complications were scrotal swelling in 7 (1.9%), wound infection in 6 (1.6%), wound dehiscence in 3 (0.8%), and transient sexual dysfunction in 31 (8.6%) patients (Fig.2). No permanent sexual function deficit was reported. The overall success rate was 97.8% with a 100% success rate on follow-up of 6 months to three years.

## DISCUSSION

Bulbar Urethral strictures are the most common type of urethral strictures prevailing in both the developing and the developed world. The most common cause of the disease is idiopathic followed by trauma. In this study, we have reported the long-term outcome and post-surgical complications experienced by patients who were surgically managed with non-transecting anastomotic bulbar urethroplasty at the Liaquat National Hospital, Karachi.

Our study reported, the incidence rates of bulbar, bulbo-membranous, and bulbo-penile strictures of 179 (50%), 111 (31%), and 68 (19%) respectively. This result ties well with previous studies wherein the bulbar urethral stricture is the most common type of urethral stricture.^6^

There are many techniques that are being used to manage the bulbar urethral stricture depending upon the cause, the size of the stricture, and the scar tissue formation. There have been numerous studies to investigate the different modalities and techniques used to treat urethral stricture disease.

Some of the most commonly used procedures for very small strictures are urethral dilatation, endoscopic treatment, and urethrotomy. However, not only are these procedures severely painful but they also cause several complications including bleeding, infection, sexual dysfunction, and recurrence of the disease. Santueci et al. in his study investigating the internal urethrotomy reported that 7.5% of their patients had post-procedural bleeding, 17% reported infection and urosepsis, 1.2% had extravasation, 1% experienced incontinence and impotence, and a recurrence rate of 30% was reported.^7^ However, when comparing our results to those of Santueci et al., we reported neither bleeding nor extravasation in any of the patients. We observed no immediate perioperative complications and only 2.51% wound related complications.

In cases of traumatic bulbar strictures, where excessive scar tissue is formed, surgeons prefer the end-to-end anastomotic urethroplasty. This procedure is reserved for the repair of the 1–2 cm-long bulbar urethral strictures. Previous literature has supported the non-transecting approach of anastomotic bulbar urethroplasty. This is because it is necessary to maintain the bulbar urethral arterial supply to avoid post-operative sexual dysfunction.^8^,^9^

In the present study, patients with small bulbar urethral strictures underwent non-transecting anastomotic bulbar urethroplasty which is comparatively new technique introduced by Dr. Mundy and his team.^10^ The main indications of this procedure are idiopathic small bulbar urethral strictures and bulbar strictures that are either idiopathic or have occurred after transurethral operation for benign prostate hyperplasia. In the present study, only patients with small bulbar urethral strictures were included, the mean stricture length in our study was found to be 1.2 cm with the smallest stricture of 0.5 cm and the largest being 2.5 cm long. While exploring the causes of the strictures, we found that 154 (43%) were idiopathic, 78 (22%) had a history of trauma, 82 (23%) had strictures due to an iatrogenic injury, while 44 (12%) had inflammatory causes. These clinical findings are well in line with other reports from the region.^11^,^12^

However, in contrast to our findings, Hussain et al. reported, trauma to be the most frequent cause of bulbar urethral strictures (49.5%) in the same geographic region. This can be explained by the fact that with advances in health care management, morbidity and mortality associated with trauma has significantly reduced in developing countries such as ours.^13^

The surgical technique used in the present study is inspired by the technique introduced by Dr. Mundy in 2005.5 After preoperative evaluation, patient is prepared for surgery. The bulbar urethra was first mobilized circumferentially and incised dorsally. The diseased portion of the bulbar urethra was excised cautiously. Then anastomosis was performed with a fine suture. The mean operative time in the study was 41 ± 12.65 minutes. The intraoperative blood loss was reported to be less than 100 ml. No intraoperative complications were reported. Patients were recommended to take bed rest of 24 hours at least with two days of hospitalization. These findings support the findings reported by Andrich et al. who associated the non-transecting anastomotic urethroplasty with early recovery, less surgical trauma, with few or no complications.^14^

Upon comparison, the technique of transecting the bulbar urethra for small strictures reported a frequency of 18-23%^15^,^16^ sexual dysfunction postoperatively whereas, our study reported only 31 (8.6%) patients with transient erectile dysfunction at 8th week of follow-up. This number further reduced to 9 (2.51%) at 24th week of follow-up. There was full recovery of sexual function at long-term follow-up of three years.

In a recent retrospective study, David et al. compared transecting and non-transecting techniques, reporting no significant difference in success rates of 93.8% for transecting while 97.9% for non-transecting technique with a p-value of 0.18.^17^ However, 14.3% of patients who were managed with transecting urethroplasty reported reduced sexual function while, with non-transecting technique only 4.3% of patients reported sexual dysfunction (p-value = 0.008). In short, transecting urethroplasty was significantly associated with sexual dysfunction (p = 0.01).

In the current study the main post-surgical complications included scrotal swelling in 7 (1.9%), wound infection in 6 (1.6%), wound dehiscence in 3 (0.8%), and transient sexual dysfunction in 31 (8.6%) patients. These findings are in line with other studies.^18^,^19^

In the present study, a success rate of 97.8% was reported which supports previous literature.^17^-^20^ Our findings indicate that the non-transecting technique has superiority over the transecting technique as it is less likely to cause any permanent sexual dysfunction among patients. A follow-up of more than 3 years can be established to further observe the long-term postoperative complications.

### Limitation of the study

Due to a restricted sample size from a single tertiary care center, the findings of our could not be generalized to a larger population therefore limiting the significance of the current study. We recommend that future studies should include a diversified and a larger sample size.

## CONCLUSION

Non-transecting anastomotic bulbar urethroplasty is a comparatively new and versatile technique for urethral stricture disease. However, our study reported promising long-term outcome with no significant intra and postoperative complications. We highly recommend urologist to consider this technique for selective patients with smaller bulbar strictures with diverse etiologies.

### Authors Contribution:

**IIM:** Responsible and accountable for the accuracy or integrity of the work.

**AA & IIM:** Conceived, designed & editing of manuscript.

**SFA:** Did data collection, statistical analysis and manuscript writing.

**AA & IIM:** Did review and final approval of manuscript.
